# Synthesis and hypoglycemic efficacy assessment of epigallocatechin gallate-selenium nanoparticles (EGCG-Se NPs) nanocomposites

**DOI:** 10.7717/peerj.20939

**Published:** 2026-03-30

**Authors:** Huiqing Ma, Lijia Sun, Huaibo Yuan, Wenhong Liu

**Affiliations:** 1 School of Biological and Food Engineering, Hefei University of Technology, Anhui, Hefei, China; 2 School of Chemistry and Chemical Engineering, Hefei University of Technology, Anhui, Hefei, China

**Keywords:** Epigallocatechin gallate, Selenium nanoparticles, Hypoglycemia, α-Amylase, α-Glucosidase

## Abstract

The synthesis of Selenium nanoparticles (Se NPs) using plant extracts has been widely recognized for many advantages. In this study, Epigallocatechin gallate (EGCG) was used as the reducing agent for sodium selenite to obtain Epigallocatechin gallate-Selenium nanoparticles (EGCG-Se NPs) whose hypoglycemic potential was evaluated. The Ultraviolet-visible(UV-Vis) spectra, Fourier Transform Infrared Spectroscopy (FTIR) profiles, and X-ray Photoelectron Spectroscopy (XPS) analysis of EGCG-Se NPs all confirmed the Se-O coordination bonds between Se NPs and EGCG, which is conducive to improving the stability of the molecular structure of the complex. The ellipsoidal structure (20–90 nm) and amorphous state of EGCG-Se NPs were characterized *via* X-ray Diffraction (XRD) and Scanning Electron Microscopy (SEM). Dynamic light scattering (DLS) and zeta potential analyses confirm that EGCG-SeNPs exhibit sustained colloidal stability under physiologically relevant buffer conditions (pH 7.2–7.4). The EGCG-Se NPs have a significant dose-responsive inhibitory effect on α-amylase (6.1–15.9%) and α-glucosidase (2.8–5.7%). *In vitro* studies have demonstrated the potential of this complex for both the supplementation of selenium and blood glucose regulation, warranting further *in vivo* validation.

## Introduction

Selenium (Se) is one of the essential trace elements for animals, plants and human beings. It is closely associated with the onset and progression of diabetes mellitus (Zhao et al., 2022). Se can enhance insulin signal transduction by regulating the activity of protein kinases, thereby increasing the sensitivity of cells to insulin and improving insulin resistance (Gao, 2023; Wang et al., 2024). Comparative studies reveal that organic Se achieves a 70–90% absorption rate under physiological conditions, while selenite absorption rarely exceeds 60% (Huang et al., 2023). Selenium nanoparticles (Se NPs) have been demonstrated to possess superior degradability and bioavailability (Deng et al., 2019; Sakr, Korany & Katti, 2018; Yin et al., 2017). Se NPs synthesis is predominantly achieved through physical, chemical, or biosynthetic routes. The physical method is defined as the application of mechanical forces to solid raw materials to alter particle size, yet it is characterized by low product purity and poor control over particle size distribution. While the chemical method allows for size control through adjustments of reaction parameters, it requires the addition of surfactants or stabilizers during synthesis. However, residual dispersing agents required in large quantities to prevent nanoparticle aggregation may lead to secondary pollution and hinder substrate absorption (Thatyana et al., 2023). Biological methods, particularly those utilizing plant extracts, have garnered attention due to their cost-effectiveness and environmental friendliness. This approach serves as a competitive alternative to microbial systems, which often involve complex cultivation and purification processes (El-Sayyad et al., 2020; Mulla et al., 2020). Plant-mediated synthesis significantly reduces the use of hazardous solvents and waste generation while eliminating the need for specialized culture conditions (Bisht, Phalswal & Khanna, 2022). However, the selection of appropriate plant extracts remains crucial, as it directly determines the physicochemical properties and biomedical applicability of the synthesized selenium nanoparticles. This underscores the necessity for a controlled and environmentally friendly synthesis strategy to ensure both reproducibility and functionality, a challenge that the present study aims to tackle by employing standardized plant polyphenols.

Epigallocatechin gallate (EGCG), extracted from tea leaves, is the most abundant catechin in green tea, accounting for approximately 50% of the total catechins in green tea polyphenols (Capasso et al., 2025; Dai et al., 2022). The molecular structure is characterized by three aromatic rings, containing multiple phenolic hydroxyl (-OH) groups, which confer strong reducing capacity. It can be used to reduce selenium salts (Escudero et al., 2025). Furthermore, EGCG has been demonstrated to inhibit the enzymatic activities of α-glucosidase and α-Amylase, effectively delaying carbohydrate hydrolysis and thereby exhibiting significant hypoglycemic potential (Kothari et al., 2024a). The stability and bioactivity of EGCG have been significantly enhanced through nano-encapsulation technology (Wang et al., 2014). Following nanoparticle formation, their surfaces can be modified with various agents, such as uptake enhancers and polyethylene glycol (PEG) (Chiu et al., 2010). The synthesis of Se NPs is facilitated by EGCG as an eco-friendly reductant, and their integration reciprocally elevates the bioavailability of EGCG, jointly mediating glucose-lowering efficacy.

In this study, EGCG-Se NPs were synthesized using EGCG as the reducing agent and sodium selenite as the Se source. The synthesized composites were systematically characterized, followed by *in vitro* hypoglycemic activity evaluation. The experimental design aimed to validate the successful synthesis of EGCG-Se NPs, elucidate synergistic interaction mechanisms between constituent components, and assess their glucose-lowering efficacy.

## Material & Methods

### Material and instrument

EGCG was purchased from Shanghai Maclin Biochemical Technology Company Ltd. Sodium selenite and 3,5-dinitrosalicylic acid (DNS) were obtained from Shanghai Yi En Chemical Technology Company Ltd., while PEG was sourced from Shanghai Bronkon Chemical Company Ltd. α-Amylase and α-glucosidase were purchased from Shanghai Yuanye Biotechnology Company Ltd. and Beijing Solaibao Technology Company Ltd., respectively. p-Nitrophenyl-α-D-glucopyranoside (PNPG) and acarbose were both purchased from Aladdin Chemical Technologies Company. The model of the enzyme calibration is Multiskan FC Thermo. The model of the freezing dryer is FD-1A-50+.

### Methods

#### Preparation and characterization of EGCG-Se NPs

EGCG-Se NPs were synthesized following a modified method based on previously reported methods (Abdul-Razek et al., 2024; Saleh et al., 2023). Briefly, sodium selenite (10 g) was dissolved in a mixed solvent of methanol (50 mL) and water (50 mL) under stirring to obtain a 50 mM solution. After adjusting the initial pH from 10.2 to 8.6 with 1 molar hydrochloric acid (1 M HCI) (Muzolf et al., 2008), EGCG (1.32 g, molar ratio to Na_2_SeO_3_ = 1:20) was added and stirred to dissolve, followed by the addition of PEG-10000 (2 g, mass ratio to Na_2_SeO_3_ = 5:1) with continued stirring until full dissolution. The reaction mixture was sealed, kept in the dark at 25 °C, and stirred continuously for 5 days. During this period, the solution color transitioned from purple to yellow and finally to black. After 5 days, stirring was stopped and the mixture was allowed to stand undisturbed for an additional 24 h (Sentkowska & Pyrzyńska, 2022; Tarmizi et al., 2023). Most of the methanol was then removed from the mixture using a rotary evaporator under reduced pressure with a water bath temperature maintained between 40–50 °C. The remaining solution was transferred to a lyophilization vial. The product was obtained as a solid powder by freeze-drying, with an overall yield of 23.36%. Subsequently, the powder was stored in a sealed container at 0–4 °C. Prior to analysis, a weighed sample was dissolved in deionized water or phosphate-buffered saline (PBS, pH7.2–7.4) to yield the desired final concentration. The resulting suspension was vortexed for 1–2 min and then subjected to pulsed sonication (100 W, 30 s on/10 s off, 2–3 cycles). The lower pH reduced the reaction rate, making it insufficient to reach completion within a shorter timeframe (Ibrahim et al., 2024). Therefore, after adjusting the pH from 10 to 8.6, a 5-day reaction period was employed. The strong alkaline environment (pH10) was excluded because EGCG is more susceptible to degradation than other common reducing agents. Under these conditions, EGCG is readily oxidized by atmospheric oxygen, a process not sufficiently prevented by sealing alone. The described synthesis has been independently repeated three times under identical conditions (room temperature, dark, sealed, pH8.6, fixed molar/mass ratios).

EGCG and EGCG-Se NPs aqueous solutions (0.3 mg/mL in ultra-pure water) were scanned from 200–800 nm on a CARY 5,000 ultraviolet-visable (UV Vis) spectrophotometer. Fourier transform infrared (FT-IR) spectra of EGCG and EGCG-Se NPs were recorded (4,000–500 cm^−1^) on a Shimadzu 3,700 deep ultraviolet (DUV) spectrometer using KBr pellets of vacuum-dried samples. X-ray diffraction (XRD) analysis of samples was performed on an XPert Pro MPD diffractometer (Malvern Panalytical) using Cu Kα radiation (40 kV, 40 mA) (Jin et al., 2022), scanning 10−80° (2*θ*) at 10°/min (step 0.03°) at 20 °C. EGCG-Se NPs morphology was observed using a Thermo Fisher Quattro S EDS. Gold-coated samples were analyzed at 7.4 mm working distance (WD), 2.0 µm spot size (Ma et al., 2025). Elemental mapping of EGCG-Se NPs was performed on a Thermo Fisher Quattro S (EDS: EDAX ELECT PLUS) at 20 kV and 1,000× magnification. X-ray photoelectron spectroscopy (XPS) analysis of EGCG-Se NPs was performed using a Thermo Scientific Nexsa spectrometer equipped (monochromatic Al Kα source, 15 kV, 10 mA). The colloidal stability and particle size distribution of the EGCG-Se NP solution were monitored over a 72-hour period using dynamic light scattering (DLS) and zeta potential measurements (Nano ZS90 analyzer), with evaluations conducted at 24, 48, and 72 h.

#### α-Amylase and α-glucosidase inhibitory activity test

The α-amylase and α-glucosidase inhibitory activity of the synthesized EGCG-Se NPs was determined according to a modified method based on the method previously reported by Kothari et al. (2024b). Briefly, 200 µL of α-amylase solution (prepared by dissolving 0.05 g of α-amylase in 100 mL of deionized water, 3 U/mL) was premixed with 50 µL of EGCG-Se NPs suspension at different concentrations (0.2, 0.4, 0.6, 0.8, and 1 mg/mL) in a 1.5 mL EP tube, followed by vortexing for 30 s at room temperature(25 °C). Then, 250 µL of starch substrate solution (0.5 mg/mL, prepared in 0.05 M phosphate buffer, pH 6.6) was added to initiate the reaction. After incubation at 37 °C for 15 min, 250 µL of DNS reagent was added to stop the reaction, and the mixture was heated in a boiling water bath (100 °C) for 15 min. Finally, 200 µL from each tube was transferred to a 96-well plate, and the absorbance was measured at 540 nm using a microplate reader. For the α-glucosidase inhibition assay, 200 µL of α-glucosidase solution (0.05 g α-glucosidase dissolved in 100 mL deionized water, 0.3 U/mL) was premixed with 50 µL of EGCG-Se NPs suspension (0.2, 0.4, 0.6, 0.8, and 1 mg/mL) in a 1.5 mL EP tube, followed by vortexing for 30 s at room temperature. After the addition of 200 µL of 3 mM PNPG (in 100 mM phosphate buffer, pH 6.6), the reaction mixture was incubated at 37 °C for 30 min and then terminated by adding 200 µL of 0.1 M Na_2_CO_3_ solution. Finally, the absorbance was measured at 405 nm. In both inhibition experiments, control groups were prepared by replacing the sample with an equal volume of buffer, and acarbose was used as a positive control. The buffer used for diluting samples was phosphate-buffered saline (PBS) (1 ×). Inhibition activity was calculated using the corresponding equations (Eq. (1) for α-amylase, Eq. (2) for α-glucosidase). All experiments were performed in triplicate (*n* = 3), and data are expressed as mean ± SD.

(1) \begin{eqnarray*}I~(\%)= \frac{{A}_{540~control}-{A}_{540~sample}}{{A}_{540~control}} \times 100\end{eqnarray*}

(2) \begin{eqnarray*}I~(\%)= \frac{{A}_{405~control}-{A}_{405~sample}}{{A}_{405~control}} \times 100\end{eqnarray*}

#### Statistical analysis

Data are presented as mean ± standard deviation (SD) derived from three independent synthesis batches. The statistical analysis was performed by one-way ANOVA using SPSS version 27.0. The differences between experimental groups were considered significant when the *P* value was < 0.05 (*P <0.05).

## Results

### UV-Vis and FTIR analysis

**Figure 1:**
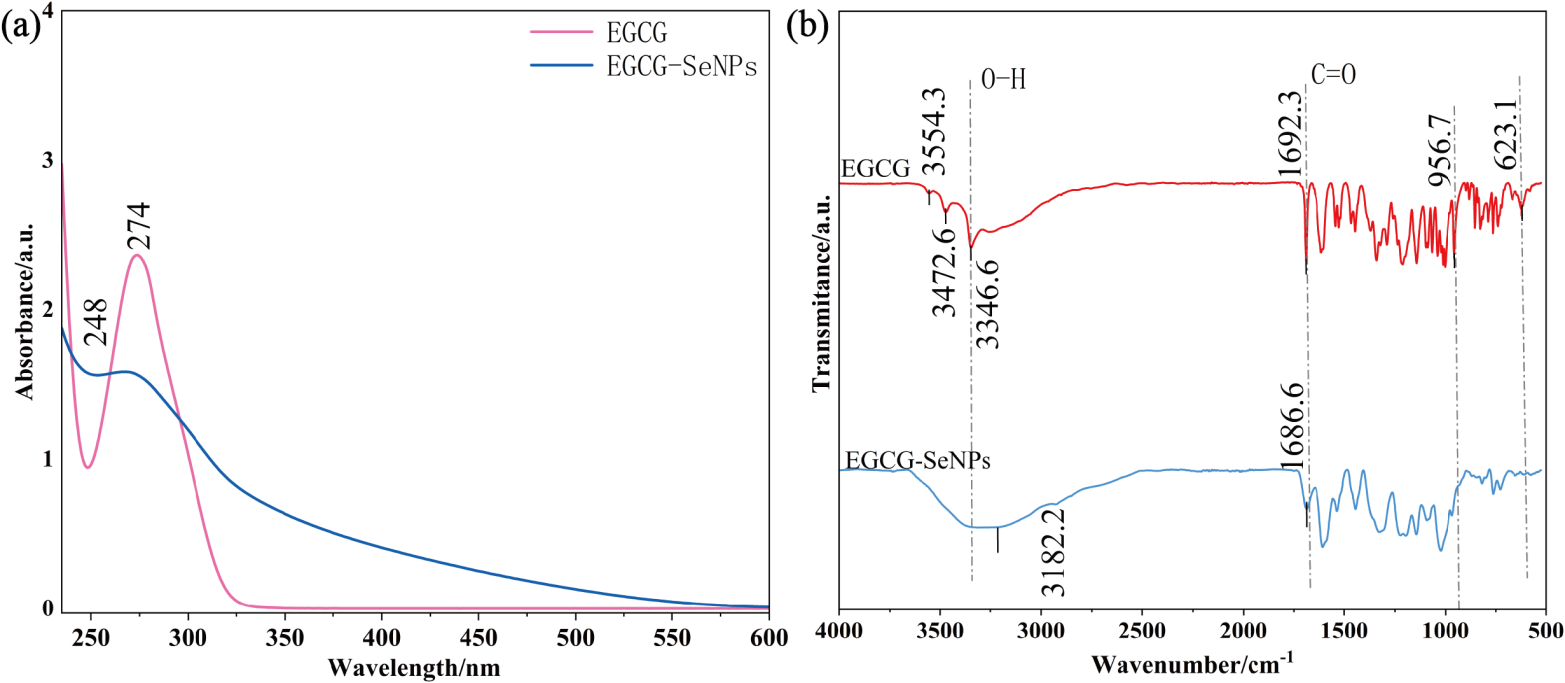
The absorption wavelengths of EGCG and EGCG-Se NPs in UV-vis and FT-IR. (A) UV-Vis spectra of EGCG and EGCG-Se NPs. (B) FT-IR spectra of EGCG and EGCG-Se NPs.

UV-Vis spectroscopy was used to analyze the molecular structural changes of EGCG-Se NPs and EGCG. There is an absorption peak at 274 nm in Fig. 1A, which is attributed to the *π*-conjugated electronic system of its aromatic rings and the electron-donating substituents on the benzene skeleton (Yu & Li, 1994). Compared to pure EGCG, EGCG-Se NPs exhibit a broadened absorption band at 274 nm with reduced intensity, accompanied by a diffuse absorption profile spanning 274–500 nm. These spectral changes are attributed to new electronic interactions leading to structural reorganization, indicating substantial structural modifications in the nanocomposite. The absorption peak at 248 nm also shows a similar pattern to the above.

The FT-IR data also demonstrate that chemical structural reorganization occurred during EGCG-Se NPs formation. As is seen in Fig. 1B, there are characteristic O-H stretching vibrations of EGCG at 3,554.3, 3,472.6 and 3,346.6 cm^−1^ corresponding to free hydroxyl groups, intramolecular and intermolecular hydrogen-bonded hydroxyls respectively. In the EGCG-Se NPs complex, partial disappearance of these peaks is observed. Notably, the hydroxyl stretching vibration at 3,182.2 cm^−1^ exhibits a significant bathochromic shift compared to typical free hydroxyls (3,700–3,200 cm^−1^), indicating substantial electron density redistribution within hydroxyl groups due to chemical coordination with Se NPs (Yu & Li, 1994). This phenomenon may be attributed to the formation of coordination bonds (*e.g.*, O-Se) between the phenolic hydroxyl groups of EGCG and Se atoms, which reduces the vibrational frequency of the O-H bonds. Furthermore, the absorption peak corresponding to the C=O stretching vibration in EGCG was observed at 1,692.3 cm^−1^. Upon Se incorporation, this peak shifted to 1,686.6 cm^−1^ with reduced intensity, likely due to the formation of Se-O coordination bonds between the carbonyl oxygen and Se atoms. The formation of coordination bonds alters the electron distribution between atoms at both ends of the chemical bond, thereby modifying the transient dipole moments during vibrational modes, which directly leads to changes in infrared absorption intensities (Meng et al., 2024). Notably, within the 1,500–500 cm^−1^ spectral region, EGCG-Se NPs retained the fundamental skeletal structure of EGCG. However, the absorption band intensities were attenuated by coordination interactions. The characteristic peaks observed in EGCG at 965.7 cm^−1^ and 623.1 cm^−1^ disappeared in the EGCG-Se NPs spectrum. The spectral region spanning 1,330–667 cm^−1^, known as the fingerprint region, exhibits high sensitivity to minor structural modifications. Significant alterations observed in this region provide conclusive evidence for structural transformations during synthesis. We hypothesize that these spectral changes likely originate from Se-O coordination bonds formed between Se and oxygen atoms (*e.g.*, phenolic hydroxyl or ether oxygens), which restrict vibrational modes of the aromatic rings and suppress characteristic absorption peaks.

### X-ray diffraction, EDS and SEM analysis

**Figure 2:**
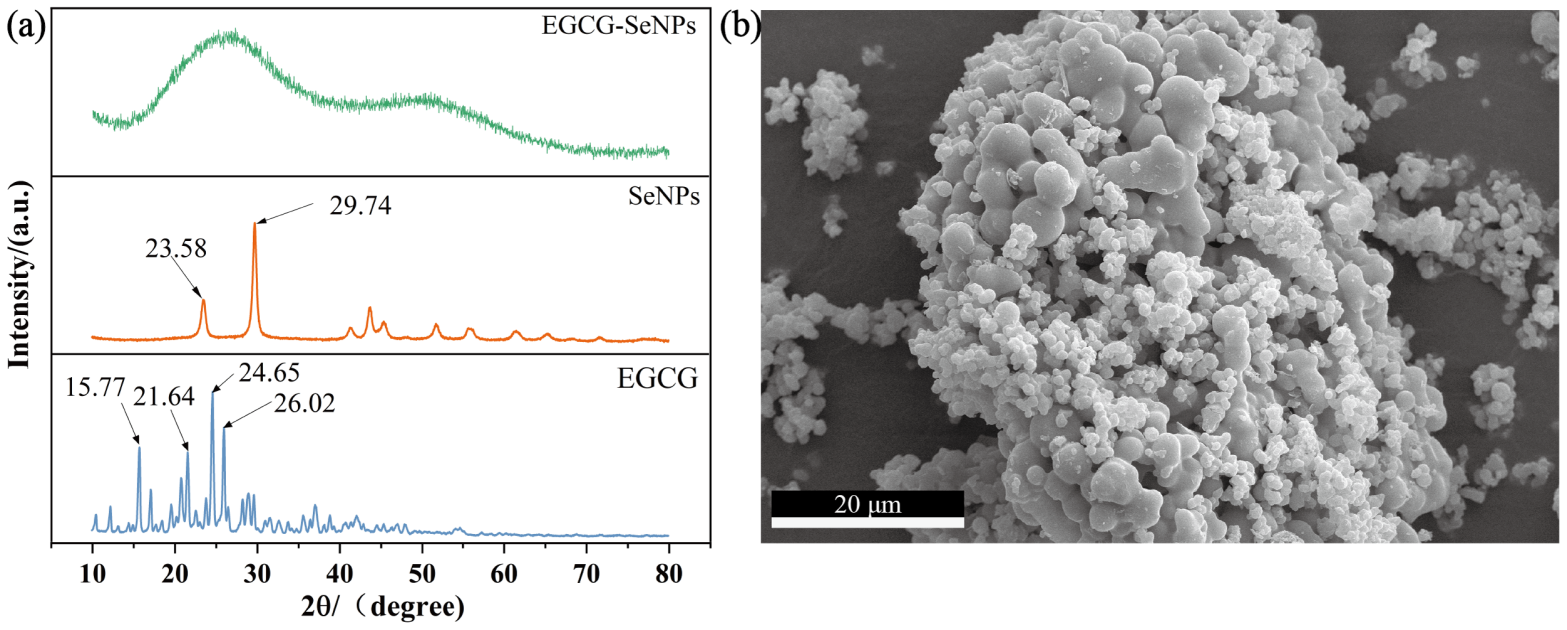
The XRD intensities of EGCG, Se NPs and EGCG-Se NPs, as well as the structure of EGCG-Se NPs. (A) The XRD spectra of EGCG, Se NPs and EGCG-Se NPs, and (B) SEM image of EGCG-Se NPs.

**Figure 3:**
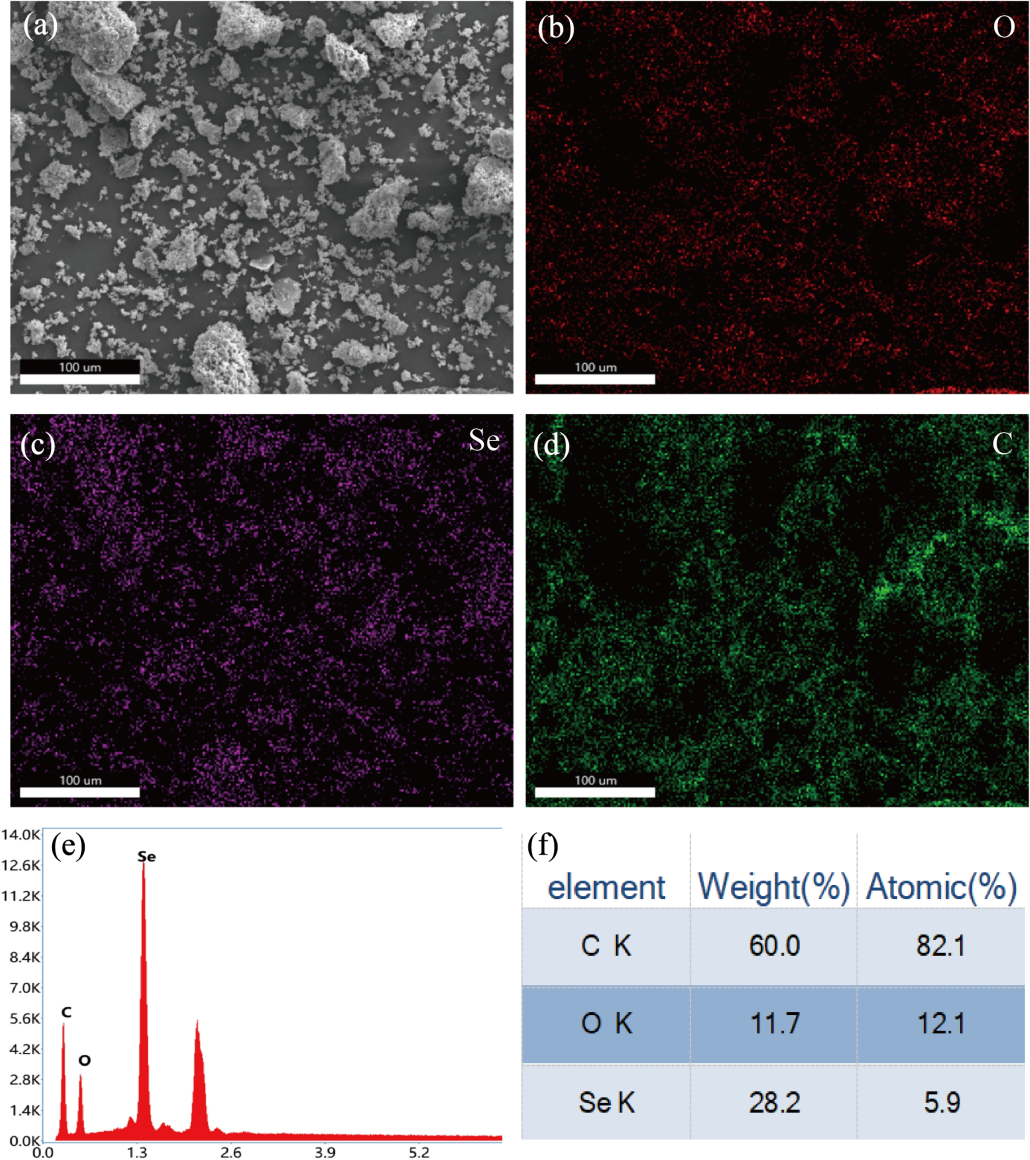
The EDS elemental mapping and quantitative analysis of EGCG-Se NPs. (A–D) The EDS elemental mapping of EGCG-Se NPs. (E, F) EDS quantitative analysis of EGCG-Se NPs.

XRD analysis was performed to compare the diffraction patterns of pure EGCG, Se NPs and EGCG-Se NPs (Panahi-Kalamuei et al., 2015; Tong et al., 2022). As it can be seen from Fig. 2A, pure EGCG has numerous characteristic peaks at 2*θ* of 15.77°, 21.64°, 24.65°, 26.02°, suggesting that EGCG had high crystalline structure (Tong et al., 2022). In the XRD pattern of EGCG-Se NPs, two broad diffraction peaks are observed, and no sharp Bragg’ peaks were present. A prominent broad peak centered at 2*θ* = 20–40° exhibits higher intensity and increased broadening compared to the characteristic peaks of pure EGCG. These features suggest partial retention of the host lattice derived from EGCG, accompanied by subtle structural modifications. The XRD pattern of EGCG combined with Se has no visible sharp peaks, indicating that a new complex was formed, and EGCG had changed from crystalline to amorphous structure (Hu, Wang & Wu, 2021; Lan et al., 2020). This phenomenon arises from the encapsulation of Se by EGCG, which significantly reduces the particle size to the nanoscale range (Gunti, Dass & Kalagatur, 2019). However EGCG is composed of hydroxyl, carbonyl, and other functional groups, which are characterized by high electronic density or coordination ability (Wen, Hui & Chuang, 2021). Another diffraction peak is observed in the 2*θ* range of 40−55° with lower intensity. This newly observed peak, absent in pure EGCG, indicates bond cleavage within EGCG molecules. The structural disruption is triggered by Se incorporation, resulting in the formation of a novel crystalline phase. These structural modifications align with prior UV-Vis and FT-IR spectroscopic evidence of coordination bonding. Comprehensive analysis reveals that EGCG-Se NPs formation involves both physical encapsulation (EGCG coating on Se NPs) and chemical interactions (Se-O coordination bonds), with all resultant composites exhibiting amorphous characteristics (Guo et al., 2024; Tang et al., 2021). Figure 2B reveals that EGCG-Se NPs form non-uniform ellipsoidal aggregates. The particle size ranges between 20 and 90 nanometers. The EDS elemental mapping in Figs. 3A–3D can confirm that homogeneous dispersion of Se throughout the composite. The EDS quantitative analysis reveals a Se atomic percentage of 5.9% in Figs. 3E–3F. Carbon and oxygen, predominantly derived from EGCG, account for 82.1% and 12.1%, respectively. These values confirm EGCG as the primary organic constituent, with Se NPs likely anchored through physicochemical interactions.

### XPS analysis

**Figure 4:**
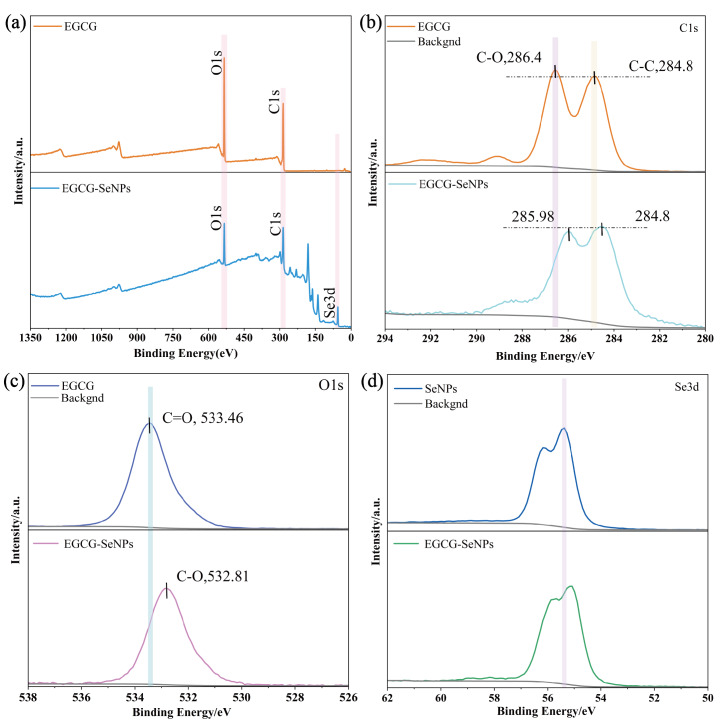
The elemental composition and chemical state of EGCG-Se NPs were studied. (A) The XPS survey spectra of EGCG and EGCG-Se NPs. (B) the C1s spectra of EGCG and EGCG-Se NPs. (C) the O1s spectra of EGCG and EGCG-Se NPs. (D) The Se3d spectra of Se NPs and EGCG-Se NPs.

XPS analysis was conducted to investigate the surface elemental composition and chemical states of EGCG-Se NPs, aiming to validate the formation of chemical bonds (*e.g.*, Se-O coordination) during the synthesis process. In Fig. 4A The survey spectrum confirmed the presence of C, O, and Se as the predominant elements, with no impurities detected. As shown in Fig. 4B, the C1s spectrum of EGCG-Se NPs exhibits a noticeable chemical shift compared to EGCG, accompanied by a reduced peak area ratio of C-O to C-C bonds, indicating decreased C-O bond intensity. Notably, in the O1s region, the binding energy of EGCG-Se NPs shifts from 533.46 eV (C=O in EGCG) to 532.81 eV (C-O) in Fig. 4C, representing a 0.65 eV decrease. In the Se 3d region, slight shifts in the XPS peaks were observed between Se NPs and EGCG-Se NPs in Fig. 4D. Deconvolution of the Se 3d spectrum resolved two peaks at 55.48 eV and 55.28 eV, assigned to Se^0^ (elemental Se).

### DLS and Zeta Potential analysis

**Figure 5:**
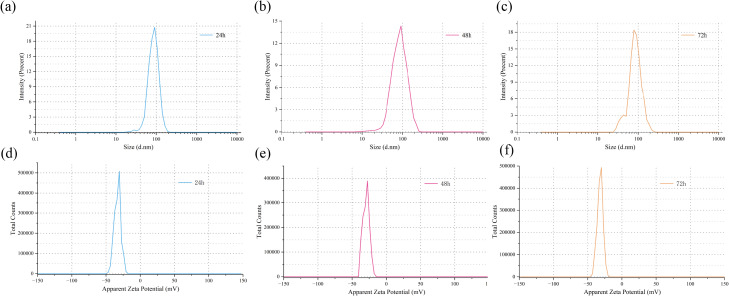
The changes in DLS and Zeta potential of EGCG-SeNPs in the buffer solution within 24–72 hours. DLS particle size distribution of EGCG-Se NPs at 24/h (A), 48/h (B), and 72/h (C). Zeta potential of EGCG-Se NPs at 24/h (D), 48/h (E), and 72/h (F). Statistical analysis by one-way ANOVA with *post hoc* tests indicated no statistically significant differences in particle size, PDI, or Zeta potential among the time points (24 h, 48 h, and 72h) (*p* > 0.05).

The colloidal stability of EGCG-Se NPs in PBS (1 ×) at 25 °C was evaluated over a period of 72h. As shown in Figs. 5A–5C, the average particle size of the produced EGCG-Se NPs by DLS were 87.56 ± 1.36 nm (24h), 97 ± 4.17 nm (48h), and 104.51 ± 13.37 nm (72h), indicating a gradual increase in particle size over time. Meanwhile, the polydispersity index (PDI) was calculated to be 0.224 ± 0.081 (24h), 0.235 ± 0.077 (48h), and 0.278 ± 0.051 (72h). Although a slight upward trend in PDI suggests a moderate broadening of the size distribution, all values remained within an acceptable range (<0.4) (Wiggers et al., 2022). Importantly, in (Figs. 5D–5F), the Zeta potential remained highly negative throughout the period, measured as -35.21 ± 1.97mV (24h), −31.57 mV ± 3.78 (48h), and −33.34 mV ± 1.3 (72h). All values exceeded the commonly referenced —−30—mV threshold associated with stable colloidal systems. Statistical analysis revealed no significant differences in size, PDI, or zeta potential across the tested time course (24–72 h). Therefore, the EGCG-Se NPs are considered stable (Lashani et al., 2024).

### Inhibitory effects of α-glucosidase and α-amylase *in vitro*

This study employed the 3,5-DNS colorimetric method to determine the α-amylase inhibitory activity. The chromogenic reaction between starch hydrolysis products and the DNS reagent was quantitatively analyzed. Based on this, systematic evaluation was performed to assess the inhibitory effects of EGCG-Se NPs (0.2−1.0 mg/mL) on the preliminary hydrolysis of carbohydrates. The PNPG method measures the amount of the product (p-nitrophenol) generated by the α-glucosidase enzyme. The ability of EGCG-Se NPs to regulate glucose release can be evaluated through this method. These two methods assess the hypoglycemic potential of the composite material by targeting distinct metabolic stages of carbohydrate digestion.

**Figure 6:**
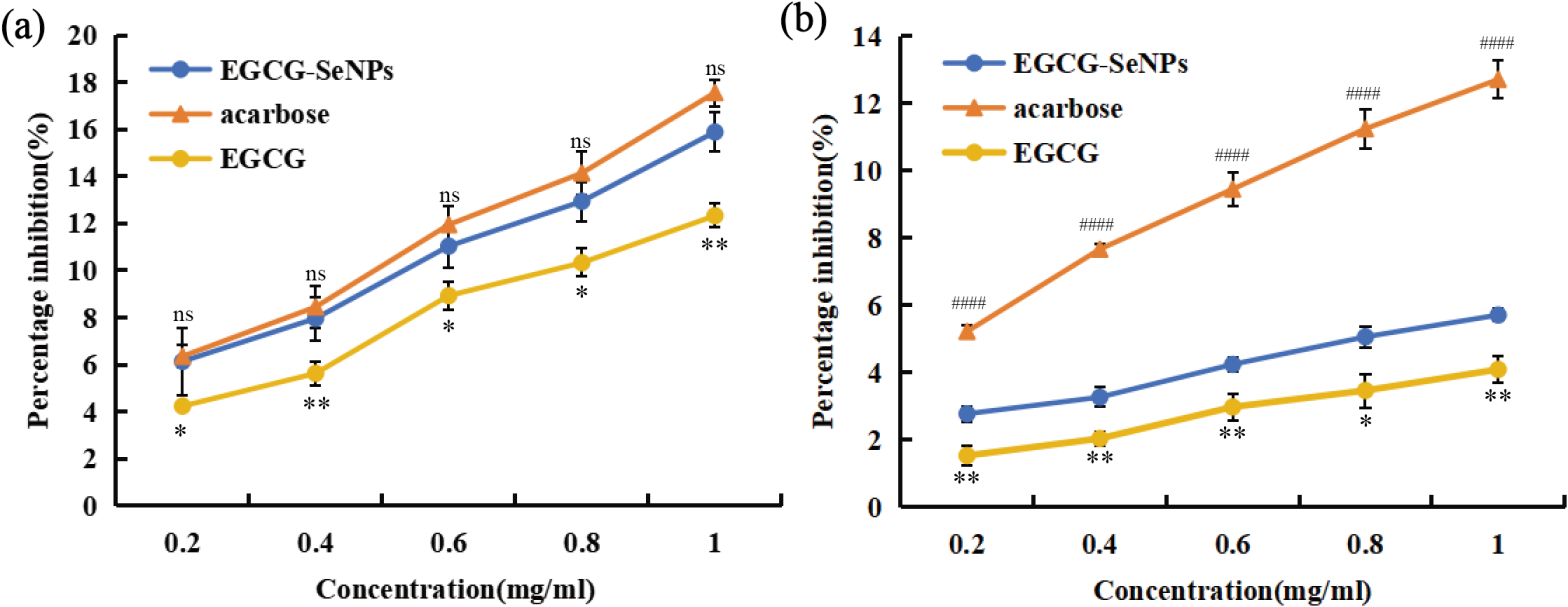
The inhibition rate of α-amylase, α-glucosidase, and the hypoglycemic effect of the studied compound. (A) The inhibition rate of α-amylase. (B) The inhibition rate of α-glucosidase. Data are presented as mean ± SD (*n* = 3 independent particle syntheses). EGCG-SeNPs *VS* EGCG: *P < 0.05, **P < 0.01; EGCG-SeNPs *VS* acarbose :^#^ *P* < 0.05, ^##^*P* < 0.01, ^####^*P* < 0.0001, ^ns^*P* > 0.05.

As shown in Fig. 6A, EGCG-Se NPs exhibited a dose-dependent increase in α-amylase inhibition, rising from 6.1 ± 1.05% to 15.9 ± 0.82%. This indicates a moderate inhibitory effect, comparable to the standard drug acarbose (no significant difference, ^#^ *p* > 0.05). It is worth noting that compared with pure EGCG (4.2 ± 0.25%–12.3 ± 0.50% inhibition at equivalent doses,^∗^ *P* > 0.05), EGCG-Se NP has a better inhibitory effect on α-amylase. Tang et al. (2021) reported an IC_5__0_ of 2.5 mg/mL for the potent inhibitor acarbose. However, the inhibition of α-amylase by EGCG-Se NPs may require concentrations higher than those tested in this study to achieve an IC_5__0_ value. To further analyze the hypoglycemic efficacy of EGCG-Se NPs, the inhibitory capacities of EGCG, EGCG-Se NPs, and acarbose against α-glucosidase were compared. As shown in Fig. 6B, EGCG-Se NPs exhibited only a weak dose-dependent response, increasing from 2.8 ± 0.23% to 5.7 ± 0.18%, which was significantly lower than the inhibitory level of acarbose (5.2 ± 1.84%-12.7 ± 0.56% inhibition at equivalent doses,^####^ *P* < 0.0001). This finding is consistent with the studies by Prasathkumar et al. (2022), Tang et al. (2021) and Tarmizi et al. (2023). However, studies demonstrated that EGCG-Se NPs exhibit a superior inhibitory effect on α-glucosidase compared to pure EGCG (1.5 ± 0.32%−4.1 ± 0.40% inhibition at equivalent doses,* *P* < 0.05). Notably, as shown in Fig. 6B, even at the highest tested concentration (1 mg/mL), the inhibition rates of EGCG-Se NPs and EGCG against α-glucosidase remained below 6%. This result clearly indicates that, within the dose range investigated in this study, both materials are not potent α-glucosidase inhibitors but rather can be defined as “mild modulators”. However, similar to their α-amylase inhibitory activity, the doses used for α-glucosidase inhibition in this study were insufficient to reveal an IC_5__0_ value Tang et al. (2021) reported an IC_5__0_ of 2.4 mg/mL for acarbose, a potent α-glucosidase inhibitor). Therefore, further studies are needed to determine the optimal concentration without compromising their potential biocompatibility.

## Discussion

This study aims to address the critical challenge of safely and stably delivering the essential trace element Se to biological systems. We successfully constructed and systematically characterized EGCG-Se NPs, exploring their dual potential for nutrient supplementation and metabolic regulation.

The significant UV-Vis spectral changes suggest that EGCG-Se NPs formation likely involves chemical bonding interactions rather than simple physical adsorption. The FT-IR spectral analysis results show three distinct changes. We hypothesize that the reason for this could be that the phenolic hydroxyl groups, carbonyl groups, and oxygen atoms on the ring of EGCG coordinate with Se. The bathochromic shift (Δ*ν*= −6 cm^−1^) can be rationalized by the electron-releasing nature of Se in low oxidation states (*e.g.*, Se^−^ or Se^0^). According to the Woodward-Fieser rules, electron-releasing substituents on aromatic ketones induce a characteristic red shift in carbonyl stretching frequencies, consistent with the observed spectral changes (Pan, 2023).

Furthermore, the irregular morphology of EGCG-Se NPs observed by XRD and scanning electron microscopy (SEM) is proposed to be a potentially beneficial feature. It is plausible that this prolonged release could underlie the observed mild inhibitory effect against digestive enzymes, thereby possibly contributing to an extended hypoglycemic activity in vitro. Additionally, by analogy with the findings of Li et al. (2022).

The decrease in the O1s region aligns with the diminished C=O stretching vibration intensity observed in FTIR spectra. The observed spectral changes can be attributed to the coordination interaction between the carbonyl oxygen (C=O) of EGCG and Se NPs, forming Se-O coordination bonds. This might be because the lone pair of electrons of Se would donate electrons to the oxygen atom through a coordination bond, increasing the electron density of oxygen and causing a decrease in the O1s binding energy. Concurrently, this electron redistribution likely reduces the polarity of the original C=O bond (alteration in dipole moment), which further explains the attenuated infrared absorption intensity. These results of XPS indicate that the system is predominantly composed of Se^0^, likely accompanied by minor surface oxidation (*e.g.*, Se^−^ species or Se-O adsorbed layer) (Ao et al., 2022). These results align with the initial hypothesis that Se predominantly exists in a low-valent state (Se^0^) with electron-donating properties. This contrasts with previous experiments where excessive reductants often induce Se^0^ oxidation to higher valence states, significantly compromising enzymatic inhibition capabilities.

Although the observed average particle size are larger than the particle size range (20–90 nm) obtained from SEM imaging, this expected discrepancy can be attributed to the hydrated state of the nanoparticles in solution or to the inherent limitations of DLS measurements. During DLS analysis, closely spaced small particles may be scanned as larger aggregates, leading to an overestimation of the hydrodynamic diameter. In summary, over the 72 h period, the EGCG-Se NPs maintained their colloidal stability and nanoscale characteristics. This provides key support for their potential feasibility in short-term biomedical applications, such as *In vitro* cell experiments, oral delivery, or topical administration.

From a real-world efficacy, α-glucosidase moderate inhibitory profile could be advantageous. Specifically, when considered as a dietary supplement, gentle modulation of enzyme activity could be beneficial, as strong inhibition of α-glucosidase may lead to incomplete carbohydrate digestion. Undigested carbohydrates can eventually be fermented by colonic bacteria, potentially causing gastrointestinal disturbances such as bloating, nausea, flatulence, and diarrhea (Cisneros-Yupanqui et al., 2023). The different results observed in this study may be attributed to methodological variations, differences in selenium nanoparticle concentration, and the source of the α-amylase used (Deepa, Mohan & Manimaran, 2022; Tang et al., 2021). Conducting a comprehensive dose-response analysis at higher concentrations, as well as performing kinetic experiments (*e.g.*, Lineweaver–Burk plots) to elucidate the inhibition type, will be important directions for future research. The main limitation of the current study lies in the lack of verification of the stability and efficacy of EGCG-Se NPs in the actual gastrointestinal fluid environment. Some nanoparticles potentially undergoing aggregation due to gastric acidity. To address these limitations, subsequent *in vivo* animal models will comprehensively evaluate their glucose-regulating effects through oral administration protocols.

## Conclusions

This study successfully synthesized Se NPs using a green synthesis method. The obtained compounds were comprehensively characterized through spectroscopic and diffraction techniques. And through *in vitro* experiments, it was verified that the compound has a hypoglycemic effect. UV-Vis and FT-IR spectra revealed significant alterations, and these molecular structure changes are critical. Structural modifications of the molecules modulate the redox potential of EGCG-Se NPs, thereby affecting their efficacy in inhibiting enzymes such as α-glucosidase and α-amylase (Capasso et al., 2025; Li et al., 2023; Liu et al., 2024; Peng et al., 2024). XRD and SEM observations revealed that EGCG-Se NPs possess an irregular shape. Analysis of the Notably, XPS showed that the oxidation state of Se remained predominantly Se^0^ with minimal changes, ensuring the retention of its bioactivity. DLS and Zeta potential results over 24–72h indicates that EGCG-Se NPs maintain good stability and homogeneity. Through *in vitro* enzyme inhibition tests, it was found that the hypoglycemic effect of EGCG-Se NPs was superior to that of pure EGCG. EGCG-Se NPs exhibited moderate inhibitory activity against α-amylase. It could delay the postprandial decomposition of starch into oligosaccharides and reduce dextrin formation. However, their inhibitory effect on α-glucosidase was limited, suggesting a relatively weak regulatory capacity over the final release of monosaccharides.

##  Supplemental Information

10.7717/peerj.20939/supp-1 Supplemental Information 1 The absorption wavelengths of EGCG and EGCG-Se NPs in UV-vis

10.7717/peerj.20939/supp-2 Supplemental Information 2 The absorption wavelengths of EGCG and EGCG-Se NPs in FTIR

10.7717/peerj.20939/supp-3 Supplemental Information 3 The XRD intensities of EGCG, Se NPs and EGCG-Se NPs, as well as the structure of EGCG-Se NPs

10.7717/peerj.20939/supp-4 Supplemental Information 4 Representative SEM micrograph of the EGCG-Se NPs

10.7717/peerj.20939/supp-5 Supplemental Information 5 The EDS elemental mapping and quantitative analysis of EGCG-Se NPs Measurements of EDS were conducted on EGCG-SeNPs, and the distributions of O/C/Se as well as their atomic weights were measured respectively.

10.7717/peerj.20939/supp-6 Supplemental Information 6 The XPS analysis was conducted on EGCG and EGCG-SeNPs respectively to observe the variation patterns of oxidation states before and after the reaction Table1: the C1s spectra of EGCG and EGCG-Se NPs. Table2: the O1s spectra of EGCG and EGCG-Se NPs. Table3: The Se3d spectra of Se NPs and EGCG-Se NPs. Table4: The XPS survey spectra of EGCG and EGCG-Se NPs.

10.7717/peerj.20939/supp-7 Supplemental Information 7 The changes in size of the three independent particles over a period of 24 to 72 hours Each data point represents the percentage of the particle within this diameter range. 24 hours, 48 hours, and 72 hours are elaborated in three separate sheets.

10.7717/peerj.20939/supp-8 Supplemental Information 8 The changes in zeta potential of the three independent particles over a period of 24 to 72 hours The ZP values of particles at different times. 24 hours, 48 hours, and 72 hours are elaborated in three separate sheets.

10.7717/peerj.20939/supp-9 Supplemental Information 9 Inhibition index of EGCG/EGCG-SeNPs/acarbose on α-amylase and α-glucosidase Each data point represents the inhibition index of EGCG/EGCG-SeNPs/acarbose on α-amylase and α-glucosidase, as well as the corresponding SD.

10.7717/peerj.20939/supp-10 Supplemental Information 10 Translation codebook

## Additional Information and Declarations

### Data Availability

The following information was supplied regarding data availability: The raw data are available in the Supplemental Files.
