# Supplementary figures and images for: Synthesis and hypoglycemic efficacy assessment of epigallocatechin gallate-selenium nanoparticles (EGCG-Se NPs) nanocomposites
Source: PeerJ. 2026 Mar 30;14:e20939. doi: 10.7717/peerj.20939 (PMC13045847; doi:10.7717/peerj.20939)

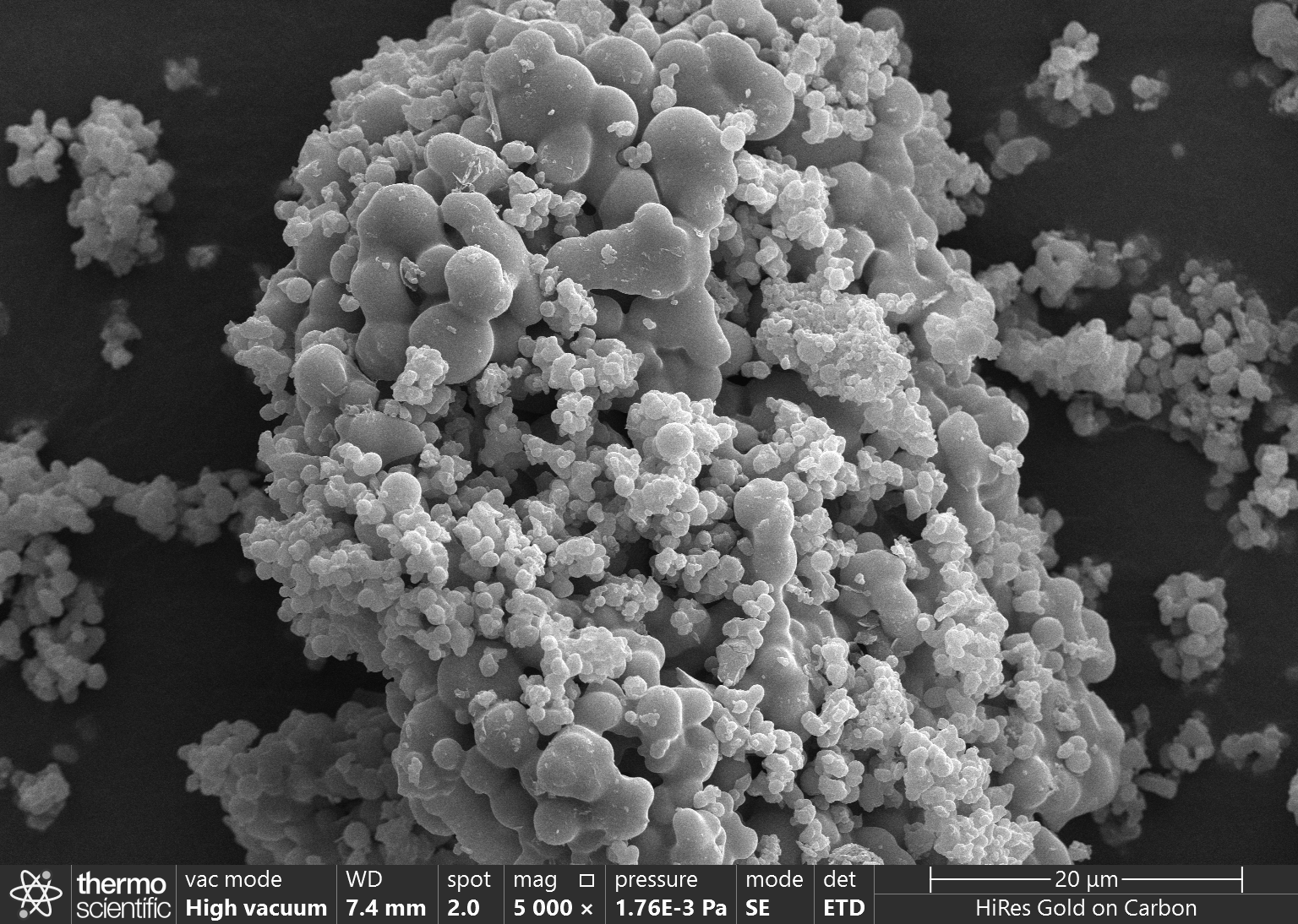

Supplement: Supplemental Information 4 [file peerj-14-20939-s004.tif]
