# Supplementary material for: Synthesis and hypoglycemic efficacy assessment of epigallocatechin gallate-selenium nanoparticles (EGCG-Se NPs) nanocomposites
Source: PeerJ. 2026 Mar 30;14:e20939. doi: 10.7717/peerj.20939 (PMC13045847; doi:10.7717/peerj.20939)
Supplement: Supplemental Information 10 [file peerj-14-20939-s010.doc]

**File: 7-size.xls**

Note on Date Format:

The column titled "Measurement Date and Time" contains timestamps formatted as "YYYY年MM月DD日 HH:MM:SS".

In this format, the Chinese characters "年", "月", and "日" correspond to the English separators "-" or "/", meaning:

"年" = year

"月" = month

"日" = day

For example:

"2025年12月15日 15:26:25" is equivalent to "2025-12-15 15:26:25".

"2026年1月16日 15:26:03" is equivalent to "2026-1-16 15:26:03".

"2026年1月16日 15:40:35" is equivalent to "2026-1-16 15:40:35".

All numerical values (year, month, day, hour, minute, second) are in the Gregorian calendar and follow the 24-hour clock.

**File: 9-in_vitro.xls**

- Sheet: [Sheet1]

- "浓度" -> "Concentration"

- "F值" -> "F-value"

- "P值" -> "P-value"

- Sheet: [Sheet2]

- "浓度" -> "Concentration"

- "F值" -> "F-value"

- "P值" -> "P-value"
